# Simulation and analysis of XCO_2_ in North China based on high accuracy surface modeling

**DOI:** 10.1007/s11356-018-2683-x

**Published:** 2018-07-22

**Authors:** Yu Liu, Tianxiang Yue, Lili Zhang, Na Zhao, Miaomiao Zhao, Yi Liu

**Affiliations:** 1 State Key Laboratory of Resources and Environmental Information System, Institute of Geographic Sciences and Natural Resources Research, Chinese Academy of Sciences, Beijing, 100101 China; 2 University of Chinese Academy of Sciences, Beijing, 100049 China; 3 Institute of Remote Sensing and Digital Earth, Chinese Academy of Sciences, Beijing, 100101 China

**Keywords:** HASM, WRF-CHEM, GOSAT XCO_2_, XCO_2_ simulation

## Abstract

As an important cause of global warming, CO_2_ concentrations and their changes have aroused worldwide concern. Establishing explicit understanding of the spatial and temporal distributions of CO_2_ concentrations at regional scale is a crucial technical problem for climate change research. High accuracy surface modeling (HASM) is employed in this paper using the output of the CO_2_ concentrations from weather research and forecasting-chemistry (WRF-CHEM) as the driving fields, and the greenhouse gases observing satellite (GOSAT) retrieval XCO_2_ data as the accuracy control conditions to obtain high accuracy XCO_2_ fields. WRF-CHEM is an atmospheric chemical transport model designed for regional studies of CO_2_ concentrations. Verified by ground- and space-based observations, WRF-CHEM has a limited ability to simulate the conditions of CO_2_ concentrations. After conducting HASM, we obtain a higher accuracy distribution of the CO_2_ in North China than those calculated using the classical Kriging and inverse distance weighted (IDW) interpolation methods, which were often used in past studies. The cross-validation also shows that the averaging mean absolute error (MAE) of the results from HASM is 1.12 ppmv, and the averaging root mean square error (RMSE) is 1.41 ppmv, both of which are lower than those of the Kriging and IDW methods. This study also analyses the space-time distributions and variations of the XCO_2_ from the HASM results. This analysis shows that in February and March, there was the high value zone in the southern region of study area relating to heating in the winter and the dense population. The XCO_2_ concentration decreased by the end of the heating period and during the growing period of April and May, and only some relatively high value zones continued to exist.

## Introduction

Global warming has drawn worldwide attention. As an important greenhouse gas, atmospheric CO_2_ concentrations can profoundly affect the trends of developments various climate scenarios, thus affecting national security and sustainable economic developments. According to the newest WMO Greenhouse Gas Bulletin (2017), globally averaged concentration of atmospheric CO_2_ rose to 403.3 ppm in 2016, compared with the 280 ppm of the preindustrial era. Therefore, to control CO_2_ emissions and reduce the effects of human activity on climate warming, understanding the spatial and temporal distributions of atmospheric CO_2_ is crucial.

A large number of ground-based observatories have been built around the world that use methods such as bottle sampling, spectroscopy, and eddy covariance to obtain information about CO_2_ concentrations and carbon fluxes. However, due to technical and financial constraints, the distribution of surface observation stations is still too sparse, and the region-scale observation capabilities are limited. In recent decades, with the development of remote sensing technology, satellite-based observations have made up for some of the deficiencies of ground-based observations. The CO_2_ concentration data retrieved from the satellite observation spectral data provide additional support for scientists studying the sources and sinks of CO_2_. SCIAMACHY is the first satellite-based detector to be sensitive to the boundary layer and acquired a large amount of observations over its 10-year orbit. Despite its lower accuracy, the data from the SCIAMACHY instrument still provides an opportunity to study the behavior of the terrestrial biosphere and climate change (Wang et al. 2011; Barkley et al. 2007). At present, the satellites in orbit that specifically make CO_2_ observations include GOSAT from Japan, OCO-2 from the USA, and TanSat from China. These satellites’ objectives are to provide global, long-term, continuous monitoring of XCO_2_ concentrations, to improve the measurement accuracies of carbon sources and sinks as well as of the regional scale CO_2_ concentrations, and to improve the understanding of their distribution characteristics and evolutions (Turner et al. 2015; Hakkarainen et al. 2016; Fischer et al. 2017). TanSat, the third carbon satellite in the world, has been active for more than a year, and the relevant research teams are working on spectral inversions of its data (Zhang et al. 2017a). The work of this paper is mainly to prepare regional scale research by TanSat data for the future, after TanSat L2 retrieval data released.

Another viable method to obtain the high resolution spatial and temporal distributions of CO_2_ concentrations is via the atmospheric chemical transport model, which uses meteorological data to represent atmosphere activities and CO_2_ fluxes to identify different emission scenarios. The chemical transport model is widely used to study CO_2_ on global and regional scales and as a comparison with observations (Shim et al. 2013; Wang et al. 2014). Additionally, by combining atmospheric chemical transport model and CO_2_ observations, atmospheric CO_2_ data assimilation has become a direct and effective approach to identify carbon sources and sinks (Peters et al. 2007; Peng et al. 2015; Tian et al. 2014). Since the model’s result is an estimation of the real world, the performance of the model is limited by the accuracy of the priori flux field and the model transport mechanism. Ground-based observations and satellite-based observations have high degrees of accuracy but have the drawbacks of limited spatial distributions and time spans (Yue et al. 2016a).

Based on the High Accuracy Surface Modeling (HASM) developed from the fundamental theorem of surfaces, the model simulation results are used as the driving fields, and the observational data as the optimal control conditions to obtain more accurate CO_2_ distribution fields. In this paper, we first simulated the CO_2_ distributions over North China using Weather Research and Forecasting - Chemistry (WRF-CHEM) as a regional atmospheric chemical transport model and evaluated the accuracy of the WRF-CHEM simulation results. Second, the CO_2_ concentration field simulated by the WRF-CHEM and GOSAT inversion data is introduced into HASM to obtain XCO_2_ concentrations. Then, the availability of HASM is verified by comparing its results with those of the classical interpolation methods commonly used in past studies (Xu et al. 2013; Liu et al. 2011). Finally, the distribution of XCO_2_ from the HASM simulation results and the changes of the monthly evolutions are discussed.

## Data and methods

### Study region

**Fig. 1:**
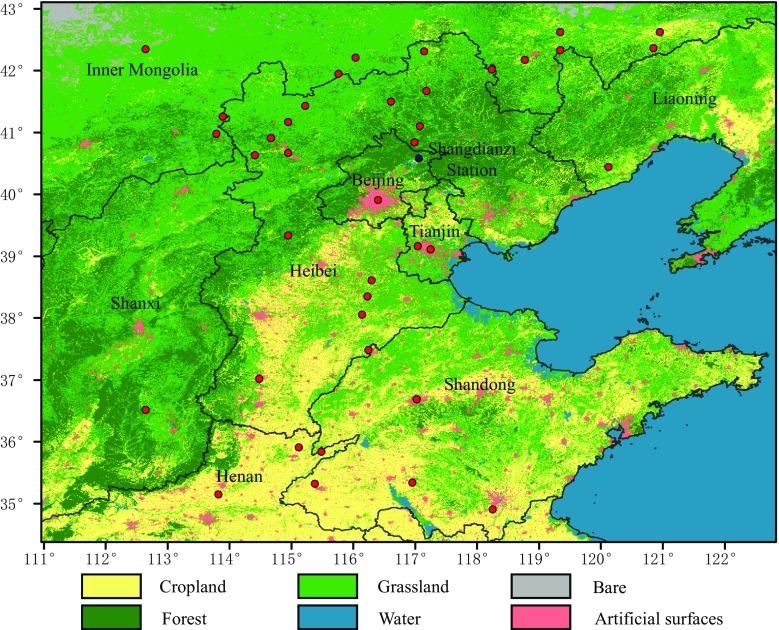
Land cover of the study region derived from ESA (European Space Agency) global land cover dataset. The red dots are the locations of the available GOSAT XCO_2_ L2 data points during the study period (from February to May 2015). The blue dot is the location of the Shangdianzi Global Atmosphere Watch (GAW) Regional Station

The study area is located in the North China, between 34.3° N~43.5° N and 111° E~121.9° E (see Fig. 1). This region incorporates the Beijing-Tianjin-Hebei region, which is a key economic zone in northern China, and Shandong Province, which is a large economically important province with the second largest population in China. In addition, part of Henan Province (which has the third largest population), Shanxi Province, Liaoning Province, and Inner Mongolia, which have a variety of underlying surfaces, including grass, forests, farmland, and cities, are also included in the study area.

### GOSAT XCO_2_ data

GOSAT (Greenhouse gases Observing SATellite) was jointly designed by the Ministry of the Environment (MOE), the National Institute for Environment Studies (NIES), and the Japan Aerospace Exploration Agency (JAXA), and was successfully launched on January 23, 2009 by the Japanese Space Agency. This satellite is the first to be used to specifically monitor the concentrations of CO_2_ and CH_4_ from space, operating at a 666 km sun-synchronous orbit with a 3-day recurrence. The TANSO-FTS on the satellite can detect the gas-absorption spectra of the reflected light in the Short Wave InfraRed (SWIR) region (0.76, 1.6, and 2.0 μm) and Thermal Infrared (TIR) band (from 5.5 to 14.3 μm) from the Earth’s surface. Using these spectra data, CO_2_ and CH_4_ can be retrieved with a footprint that measures 10.5 km along one side (Kuze et al. 2009; Kadygrov et al. 2009; Yokota et al. 2009).

In this study, the column-averaged dry-air fractions of the CO_2_ (XCO_2_) datasets released by the GOSAT project are used as the true values to verify the chemistry transport model outputs and drive the HASM. We chose the GOSAT FTS SWIR L2 data (hereafter GOSAT XCO_2_) from February to May of 2015, considering the consecutiveness of the data availability in the study area, and the data were filtered by the screening procedures described in NIES GOSAT TANSO-FTS SWIR Level 2 Data Product Format Description. The distribution of valid GOSAT XCO_2_ points is shown in Fig. 1, noting that each point may have been observed several times. The number of valid GOSAT XCO_2_ measurements from February to May are 39, 37, 30, and 33 respectively. The website containing the data is https://data2.gosat.nies.go.jp/index_en.html.

### WDCGG

The WDCGG (World Data Centre for Greenhouse Gases) is one of the data archiving and service centers under the GAW (Global Atmosphere Watch) program of WMO (World Meteorological Organization) that collects all observational data of greenhouse gases. Such as CO_2_, CH_4_, CFC, N_2_O, from 378 stations all over the world. The WDCGG was founded in October 1990 and operated at the Japan Meteorological Agency (JMA). The download website for the WDCGG data is https://ds.data.jma.go.jp/gmd/wdcgg/wdcgg.html. Based on the study period and study area, Shangdianzi Station is used as the ground observation point to verify the output of the chemistry transport model at the surface. Shangdianzi Station is located at 40.65° N, 117.12° E and is 150 km northeast of urban Beijing (see Fig. 1). This station is mainly influenced by land flux and anthropogenic emissions (Li et al. 2017).

### WRF-CHEM

The WRF-CHEM (Weather Research and Forecasting-Chemistry) is a regional air quality model developed by the National Oceanic and Atmospheric Administration (NOAA) for biomass combustion, anthropogenic emissions, chemical vapor schemes, and aerosol solutions; the model includes a trace gas transport option and a subroutine to calculate the plume lifting (Simpson et al. 1995; Sandu et al. 2003; Liu et al. 2012). The main components of the WRF-CHEM involved in this study are the WRF and greenhouse gas module.

### WRF

The WRF (Weather Research and Forecasting) model is a new generation of mesoscale weather forecasting model and was codeveloped by NCAR and NCEP. This model has a fully compressible non-static equilibrium mode and contains a wealth of physical parameterization options (Wei et al. 2016; William et al. 2008). Due to its high prediction accuracy, strong portability, fast calculation, and easy maintenance, this model is widely used in meteorological research and businesses around the world. In WRF-CHEM, the role of the WRF is to provide real-time meteorological fields for chemical modules. Because the WRF can provide a meteorological element field with a high spatiotemporal resolution, WRF-CHEM can meet the current requirements for refined forecasting. This is one of the biggest advantages of WRF-CHEM.

#### Greenhouse gas module

The greenhouse gas module of WRF-CHEM was developed by the Max Planck Institute for Biogeochemistry (Beck et al. 2011). This module is able to simulate the distributions and transports of greenhouse gases (passive tracers such as CO_2_, CH_4_, and CO) with high resolutions and is able to obtain the initial field and boundary conditions required for simulations from the global atmospheric transport model (Ahmadov et al. 2007; Diao et al. 2015; Pillai et al. 2016). The model was originally called WRFGHG (WRF Greenhouse Gas model). Starting at WRF-CHEM v3.4, the corresponding module of WRFGHG was officially included in WRF-CHEM.

In the greenhouse gas module, the Vegetation Photosynthesis Respiration Model (VPRM) is a key elemebt used to estimate the net ecosystem exchange (NEE), including the light-driven gross ecosystem exchange and the ecosystem respiration term driven by temperature.

1 $$ \mathrm{NEE}=-\uplambda \times {T}_{\mathrm{scale}}\times {P}_{\mathrm{scale}}\times {W}_{\mathrm{scale}}\times \mathrm{EVI}\times \frac{1}{\left(1+\mathrm{PAR}/{\mathrm{PAR}}_0\right)}\times \mathrm{PAR}+\alpha \times T+\beta $$

In which *λ*(μmol CO_2_ *m*^−2^*s*^−1^/(μmol PAR *m*^−2^*s*^−1^)) is the maximum quantum yield, and PAR_0_(μmol *m*^−2^*s*^−1^) is the half saturation value of the photosynthetically active radiation. EVI (Enhanced Vegetation Index) represents the ratio of the absorbed photosynthetically active radiation to the total photosynthetically active radiation. *T*_scale_, *P*_scale_, and *W*_scale_, respectively, represent the characteristics of the leaf temperatures, leaf surface characteristics, and canopy water contents. The functions of *T*_scale_, *P*_scale_, and *W*_scale_ are shown as follows:

2 $$ {T}_{\mathrm{scale}}=\frac{\left(T-{T}_{\mathrm{min}}\right)\left(T-{T}_{\mathrm{max}}\right)}{\left[\left(T-{T}_{\mathrm{min}}\right)\left(T-{T}_{\mathrm{max}}\right)-{\left(T-{T}_{\mathrm{opt}}\right)}^2\right]} $$

3 $$ {W}_{\mathrm{scale}}=\frac{1+\mathrm{LSWI}}{1+{\mathrm{LSWI}}_{\mathrm{max}}} $$

4 $$ {P}_{\mathrm{scale}}=\frac{1+\mathrm{LSWI}}{2} $$

where *T* (°C) is the temperature in atmosphere, and *T*_min_, *T*_max_, and *T*_opi_ represent the minimum, maximum, and optimum temperatures for photosynthesis. *T*_scale_ is set to 0 when the air temperature is less than *T*_min_. LSWI (Land Surface Water Index) is the moisture content of the vegetation canopy, and LSWI_max_ is the maximum LSWI value during the growing season in each grid cell. The value of *P*_scale_ depends on the growth stages of the vegetation, and for the evergreen forest, the value of *P*_scale_ is fixed at 1.0. In other cases, *T*_scale_, *P*_scale_, and *W*_scale_ range from 0.0 to 1.0. Compared with other models that treat breathing as an exponential function of temperature, VPRM reduces breathing to a linear function of temperature in which *α*(μmol CO2 m^−2^s^−1^/^°^C) and *β*(μmol CO2 m^−2^s^−1^) can be adjusted according to the observed data.

#### Running of WRF-CHEM

We use a 1°× 1 reanalysis produce, i.e., the ERA-Interim data, with time intervals of 6 h as downloaded from the European Centre for Medium-Range Weather Forecasts (ECMWF) for the initial field and boundary conditions for WRF. The total surface CO_2_ exchanges are calculated as follows:

5 $$ {F}_t={F}_{\mathrm{ant}}+{F}_R+{F}_{\mathrm{GEE}}+{F}_{\mathrm{fire}} $$

where *F*_*t*_ denotes the total CO_2_ flux. *F*_ant_ is the anthropogenic emissions obtained from the Emission Database for Global Atmospheric Research (EDGAR). *F*_*R*_ and *F*_GEE_ are the biospheric respiration and gross ecosystem exchange, respectively, and both are calculated via the VPRM in greenhouse gas module. *F*_fire_ is the biomass burning emissions provided by the Global Fire Emissions Database (GFED). The initial fields and boundary conditions of the CO_2_ concentrations from different sources are adopted from CarbonTracker2016.

The input of the VPRM module includes four kinds of data. The temperature at 2 m and the downward shortwave flux at the ground surface can be provided by the coupled WRF. LSWI and EVI are calculated from the Terra MODIS satellite level-3 land product (MYD09A1: MODIS/Aqua Surface Reflectance 8-Day L3 Global 500 m SIN Grid V006) via the VPRM-Preprocessor tool.

**Fig. 2:**
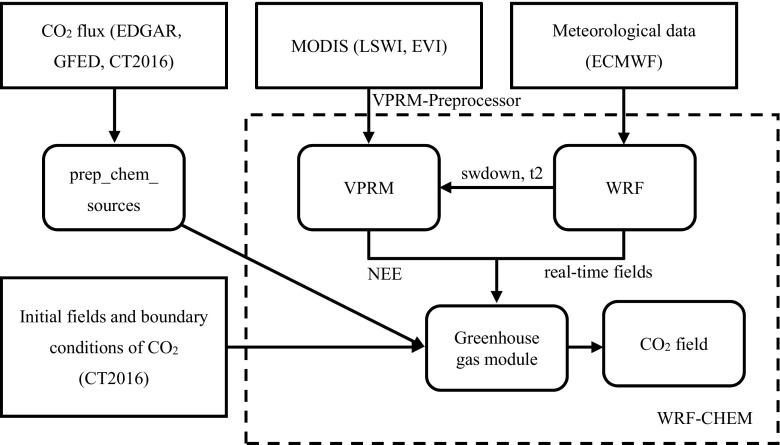
The dataset, components, and workflow involved in the operation of WRF-CHEM

The WRF-CHEM utilized is WRF-CHEM version 3.6, which is used to model from February to May 2015. During the study period, simulations were performed four times. Each simulation has a 6-h spin up for its meteorology and a 1-month run time period for the CO_2_ transport. The model domain is centered at 39.0° N, 116.5° E, with a 10 km × 10 km horizontal resolution, 35 vertical layers in the terrain-following hydrostatic-pressure vertical coordinate system from the surface to 50 hPa, and an hourly output on the Lambert projection. The chosen physical parameterization schemes are WSM 5-class microphysics scheme, RRTM longwave radiation scheme, Goddard short wave radiation scheme, revised MM5 Monin-Obukhov surface-layer scheme, unified Noah land-surface model, YSU boundary-layer scheme, and Grell 3D ensemble cumulus scheme. The dataset, components, and workflow involved in the operation of WRF-CHEM are shown in Fig. 2.

### HASM

Based on the fundamental theorem of surface, a surface is uniquely determined by the first and second fundamental coefficients. The first fundamental coefficients of a surface describe the geometric properties of the surface, by which we can calculate the lengths of the curves, the angles of the tangent vectors, the areas of regions, and the geodesics on the surface. These geometric properties and objects are called the intrinsic geometric properties and are only determined via the first fundamental coefficients of a surface, depending on measurements that we can conduct while staying on the surface itself (Toponogov 2006). The second fundamental coefficients of a surface describe the local deformations of the surface, which can be observed while staying above the surface. In other word, these are the deviations of the relevant point on the surface from the tangent plane (Liseikin 2004).

Suppose a surface *z* can be represented by a function of *x* and *y*; that is, *z* = *f*(*x*, *y*). The first fundamental coefficients, *E*, *F*, and *G*, and the second fundamental coefficients, *L*, *M*, and *N*, are defined as follow:

6 $$ E=1+{f}_x^2 $$

7 $$ F={f}_x\cdot {f}_y $$

8 $$ G=1+{f}_y^2 $$

9 $$ L=\frac{f_{xx}}{\sqrt{1+{f}_x^2+{f}_y^2}} $$

10 $$ M=\frac{f_{xy}}{\sqrt{1+{f}_x^2+{f}_y^2}} $$

11 $$ N=\frac{f_{yy}}{\sqrt{1+{f}_x^2+{f}_y^2}} $$

In which *f*_*x*_ is the first order partial derivative of the surface *z* with respect to the independent variable *x*. *f*_*xx*_ is the second order partial derivative of the surface *z* with respect to the independent variable *x*.

A similar definition applies for *f*_*y*_ and *f*_*yy*_. *f*_*xy*_ is the second order mixed partial derivative of the surface z with respect to the independent variables *x* and *y* successively.

Based on previous research, an equation set called the Gauss equation was found to relate the intrinsic curvature of the surface to the derivatives of the Gauss map, namely, the first fundamental coefficients (Eqs. 6–8) and the second fundamental coefficients (Eq. 9–11) satisfy the following equation set:

12 $$ {f}_{xx}={\Gamma}_{11}^1{f}_x+{\Gamma}_{11}^2{f}_y+\frac{L}{\sqrt{E+G-1}} $$

13 $$ {f}_{xy}={\Gamma}_{12}^1{f}_x+{\Gamma}_{12}^2{f}_y+\frac{M}{\sqrt{E+G-1}} $$

14 $$ {f}_{yy}={\Gamma}_{22}^1{f}_x+{\Gamma}_{22}^2{f}_y+\frac{N}{\sqrt{E+G-1}} $$

where

15 $$ {\Gamma}_{11}^1=\frac{GE_x-2{FF}_x+{FE}_y}{2\left( EG-{F}^2\right)} $$

16 $$ {\Gamma}_{12}^1=\frac{GE_x-{FG}_x}{2\left( EG-{F}^2\right)} $$

17 $$ {\Gamma}_{22}^1=\frac{2{GF}_y-{GG}_x-{FG}_y}{2\left( EG-{F}^2\right)} $$

18 $$ {\Gamma}_{11}^2=\frac{2{EF}_x-{EE}_y-{FE}_x}{2\left( EG-{F}^2\right)} $$

19 $$ {\Gamma}_{12}^2=\frac{EG_x-{FE}_y}{2\left( EG-{F}^2\right)} $$

20 $$ {\Gamma}_{22}^2=\frac{EG_y-2{FF}_y+{FG}_x}{2\left( EG-{F}^2\right)} $$

${\Gamma}_{11}^1$, ${\Gamma}_{12}^1$, ${\Gamma}_{22}^1$, ${\Gamma}_{11}^2$, ${\Gamma}_{12}^2$, and ${\Gamma}_{22}^2$ are the second type of Christoffel symbols and rely only on the first fundamental coefficients, *E*, *F*, and *G*, and their derivatives. In the process of solution of the Eqs. 12–14, the central difference method is used to displace the partial derivatives.

We mark the first, second, and third equations of Gauss equation set as a, b, and c. Then, HASM abc can be expressed as a constrainted least-squares approximation.

21 $$ \left\{\begin{array}{c}\min {\left\Vert \left[\begin{array}{c}A\\ {}B\\ {}C\end{array}\right]\cdot {z}^{\left(n-1\right)}-{\left[\begin{array}{c}d\\ {}q\\ {}h\end{array}\right]}^{(n)}\right\Vert}_2\\ {}S\cdot {z}^{\left(n-1\right)}=k\end{array}\right. $$

where the second equation of Eq. 21 is the constraint equation representing the sampling points information. *A*, *B*, and *C* are the coefficient matrix of the discrete equation form of the Gauss equation. *d*, *q*, and *p* are found on the right-hand side of the Gauss equation. *S* denotes the sampling matrix, and *k* denotes the sampling vector.

Equation 21 is a least-squares problem constrained by terrestrial sampling. The purpose of Eq. 21 is to confine the overall simulation error to a minimum value, while keeping the simulated value equal to the sample value at the sampling point. Taking full advantage of the sampling information is also an effective way to ensure that the iterative formulation of HASM approaches the best simulation result.

HASM can also be transferred into an unconstrained least-squares approximation:

22 $$ \left[{A}^T{B}^T{C}^T\lambda \cdot {S}^T\right]\left[\begin{array}{c}A\\ {}B\\ {}C\\ {}\lambda \cdot S\end{array}\right]{Z}^{\left(n+1\right)}=\left[{A}^T{B}^T{C}^T\lambda \cdot {S}^T\right]\left[\begin{array}{c}{d}^{(n)}\\ {}{q}^{(n)}\\ {}{p}^{(n)}\\ {}\lambda \cdot k\end{array}\right] $$

where λ denotes the weight of the sampling point values, which refers the relative importances of the sampling points in the simulated field. *λ* could be a real number or a vector, depending on whether all sampling points are equally important or if each point has its own weight.

**Fig. 3:**
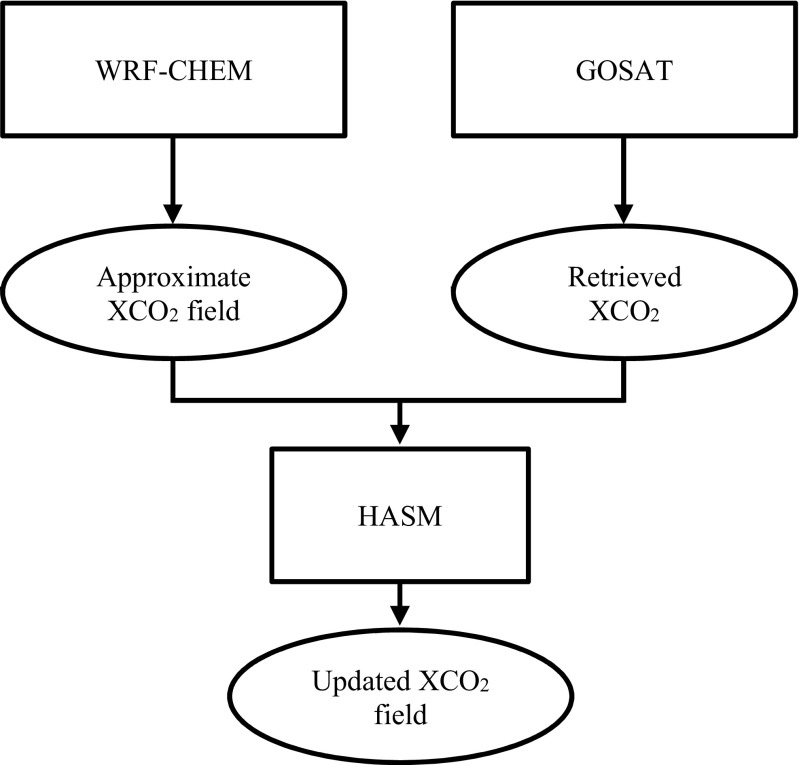
The workflow used to obtain the XCO_2_ field based on HASM

In the existing research, HASM have been applied for the study of soil properties (Shi et al., 2011, 2016), carbon storage (Wang et al., 2016; Yue et al. 2016b), and climate change (Yue et al. 2013; Zhao and Yue 2014). At a global scale, Zhao (Zhao et al. 2017a, b) and Zhang (2017b) introduced HASM for XCO_2_ simulations with plenty of sampling points. Yue et al. (2015) verified that HASM is an alternative approach to filling voids on XCO2 surfaces from satellites. In this study, we force a regional scale simulation with a scant number of sampling points to investigate the performance of HASM. Based on HASM, the workflow used to obtain the XCO_2_ field is shown in Fig. 3.

## Results and discussion

### Comparison between WRF-CHEM and WDCGG

**Fig. 4:**
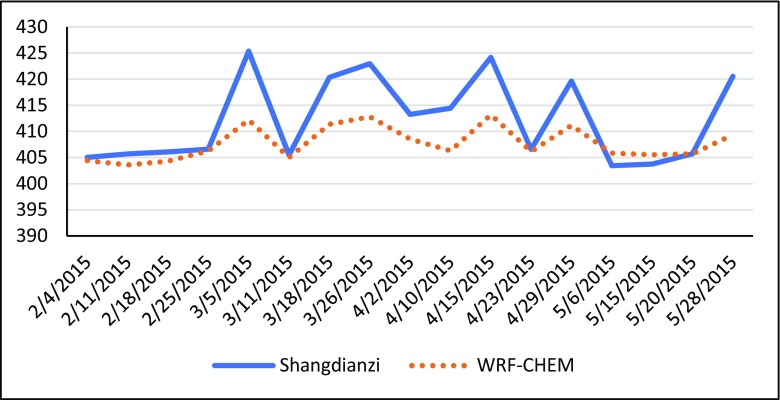
The comparison of CO_2_ values near the surface between simulations and observations. The red lines show the WRF-CHEM grid CO_2_ simulations. The blue line is the observations of Shangdianzi Station from WDCGG

Figure 4 shows the comparison between the WRF-CHEM CO_2_ data and the ground-based measurements from Shangdianzi Station during the study period. Overall, the WRF-CHEM CO_2_ simulations represent the fluctuations of the observation time series, with a high correlation coefficient of approximately 0.94. The difference between the model and observations is small in the low value range (approximately 405 ppmv), whereas the bias becomes large when the observed CO_2_ concentrations are high and the model values are lower. The reason for this difference may be that the data from the model is a mean value of a 0.1°× 0.1 grid cell. Therefore, the simulations results reflect the smooth characteristics. Due to the complexity of atmosphere and the lack of understanding of atmospheric motion, WRF includes many approximations, and the choice of many parameters is debatable. Because of these issues, WRF is unable to accurately simulate the wind field, thereby affecting CO_2_ transmission and diffusion in the atmosphere. In addition, the emission data are not very accurate, which also affects the distribution of CO_2_ concentration.

Comparison between WRF-CHEM and GOSAT

Based on the information of the dates and longitudes/latitudes in the GOSAT retrieval dataset, we extract the simulation data for the same dates and positions to compare between the simulation and GOSAT XCO2. Note that the original WRF-CHEM output is a layered CO_2_ concentration. Thus, here, WRF-CHEM XCO_2_ is calculated based on the following function:

23 $$ {\mathrm{XCO}}_2=\sum \limits_{i=1}^N{h}_i{u}_i $$

where *N* is the number of layers in the original WRF-CHEM output, *u*_*i*_ presents the CO_2_ concentrations as each layer, and *h*_*i*_ presents the pressure weighting function which relates the layered CO_2_ concentration to the profile-weighted average (Connor et al. 2008).

24 $$ {h}_i=\left|\left(-{p}_i+\frac{p_{i+1}-{p}_i}{\ln \left(\frac{p_{i+!}}{p_i}\right)}\right)+\left({p}_i-\frac{p_i-{p}_{i-1}}{\ln \left(\frac{p_i}{p_{i-1}}\right)}\right)\right|\frac{1}{P_{\mathrm{surf}}} $$

where *p*_*i*_ is the pressure on each level, and *p*_surf_ is the surface pressure. For the upper or lower boundary layers, the function retains only the left or right items within the absolute operator.

The basic statistics of the XCO_2_ from the WRF-CHEM and GOSAT retrieval data are shown in Table 1. In the 4-month study period, the simulated minimums are approximately 4–5 ppmv higher than those of the GOSAT XCO_2_, and the average of the simulations is also approximately 2–3 ppmv higher. Considering XCO_2_ is the general representation of the CO_2_ content from the surface to the top of atmosphere, and the lower simulated values of the surface CO_2_ concentration are shown in Fig. 4, the higher simulated values of XCO_2_ from WRF-CHEM may come from excess CO_2_ simulated in the troposphere. The simulated variances of each month in Table 1 are less than the observed variances, which is a common problem for transmission models (Lei et al. 2014). The correlation coefficients of WRF-CHEM XCO_2_ and GOSAT XCO_2_ are between 0.4 and 0.78, being highest in February and lowest in May.

**Table 1:** Basic statistics of XCO_2_ from WRF-CHEM and GOSAT

	2015.02		2015.03		2015.04		2015.05	
	WRF-CHEM	GOSAT XCO_2_	WRF-CHEM	GOSAT XCO_2_	WRF-CHEM	GOSAT XCO_2_	WRF-CHEM	GOSAT XCO_2_
Max (ppmv)	404.41	405.35	403.87	406.36	405.35	404.71	404.97	403.22
Min (ppmv)	400.92	396.43	401.58	395.60	402.97	398.09	402.01	398.27
Mean (ppmv)	402.17	400.21	402.41	399.59	403.84	400.65	403.83	400.87
Variance	1.04	2.74	0.57	2.96	0.72	1.71	0.66	1.31
Correlation coefficient	0.78		0.51		0.56		0.40	

From Fig. 4 and Table 1, WRF-CHEM has a certain degree of ability to simulate regional CO_2_ concentrations. However, due to the limited emission inventories and the performances of the models, the simulation accuracies remain to be improved. In the next chapter, we adopt HASM to achieve our goal, such that the CO_2_ concentrations of the WRF-CHEM model are taken as driving fields, and GOSAT XCO_2_ is used as the accuracy control conditions.

### Comparison with the Kriging and IDW methods

Taking the simulated XCO_2_ fields from WRF-CHEM and the retrieval data from GOSAT as the driving field and accuracy control conditions, respectively, we operate HASM to update the XCO_2_ field. Meanwhile, as the classic interpolation methods, Kriging and IDW methods are also involved in the experiments to compare with HASM. The XCO_2_ data is calculated on a monthly basis due to limited retrieval data. For each month, we organize the cross-validation test, which means that one data point in GOSAT XCO_2_ is selected as the test point, and the remaining points are used to drive the three methods. After this, the simulation values are extracted for the test points.

**Fig. 5:**
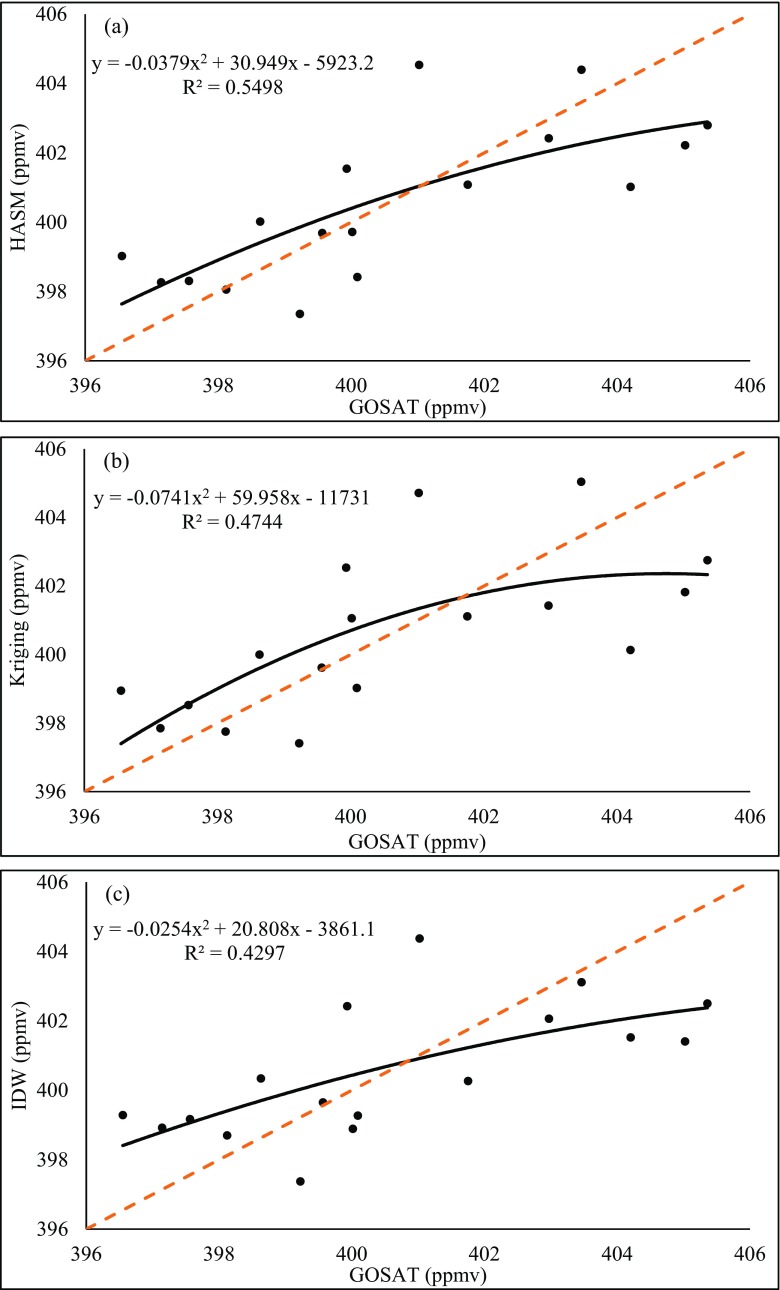
Comparison of the three methods and GOSAT retrieval data on 2015.02. **a** HASM. **b** Kriging. **c** IDW

**Fig. 6:**
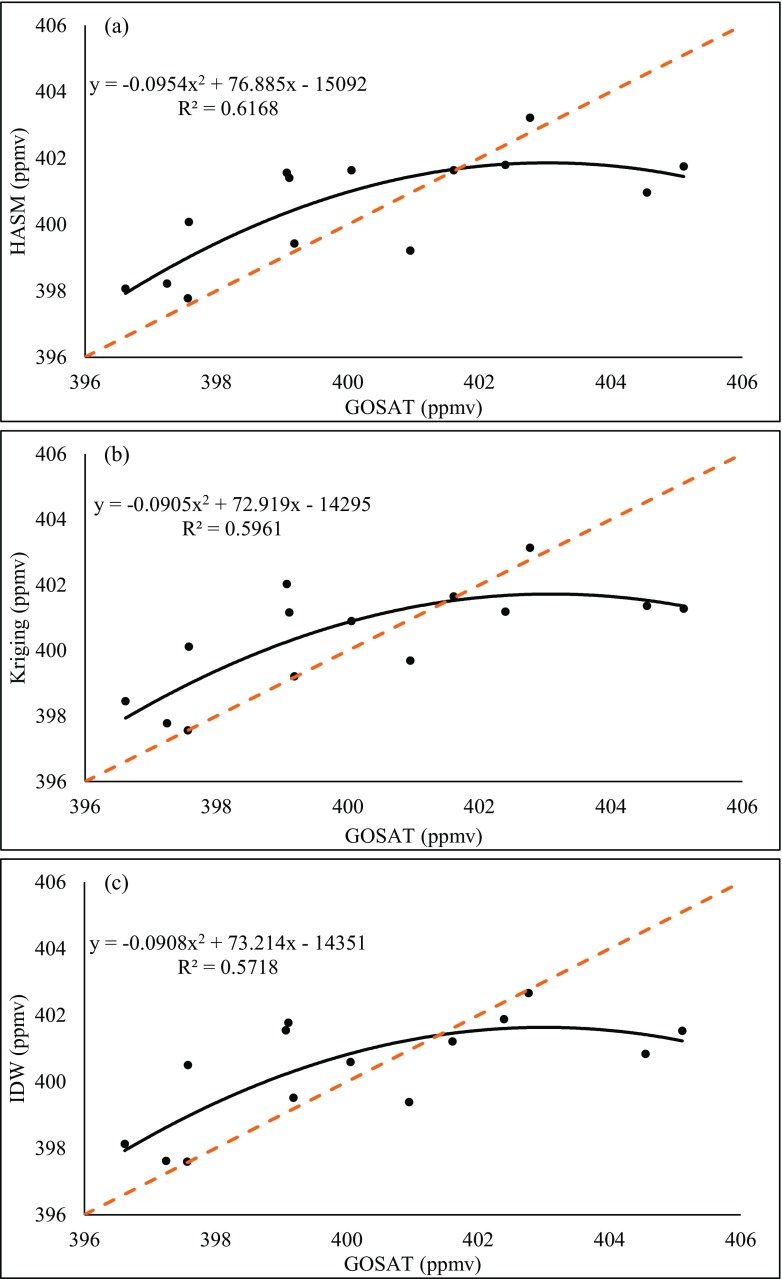
Comparison of the three methods and GOSAT retrieval data on 2015.03. **a** HASM. **b** Kriging. **c** IDW

**Fig. 7:**
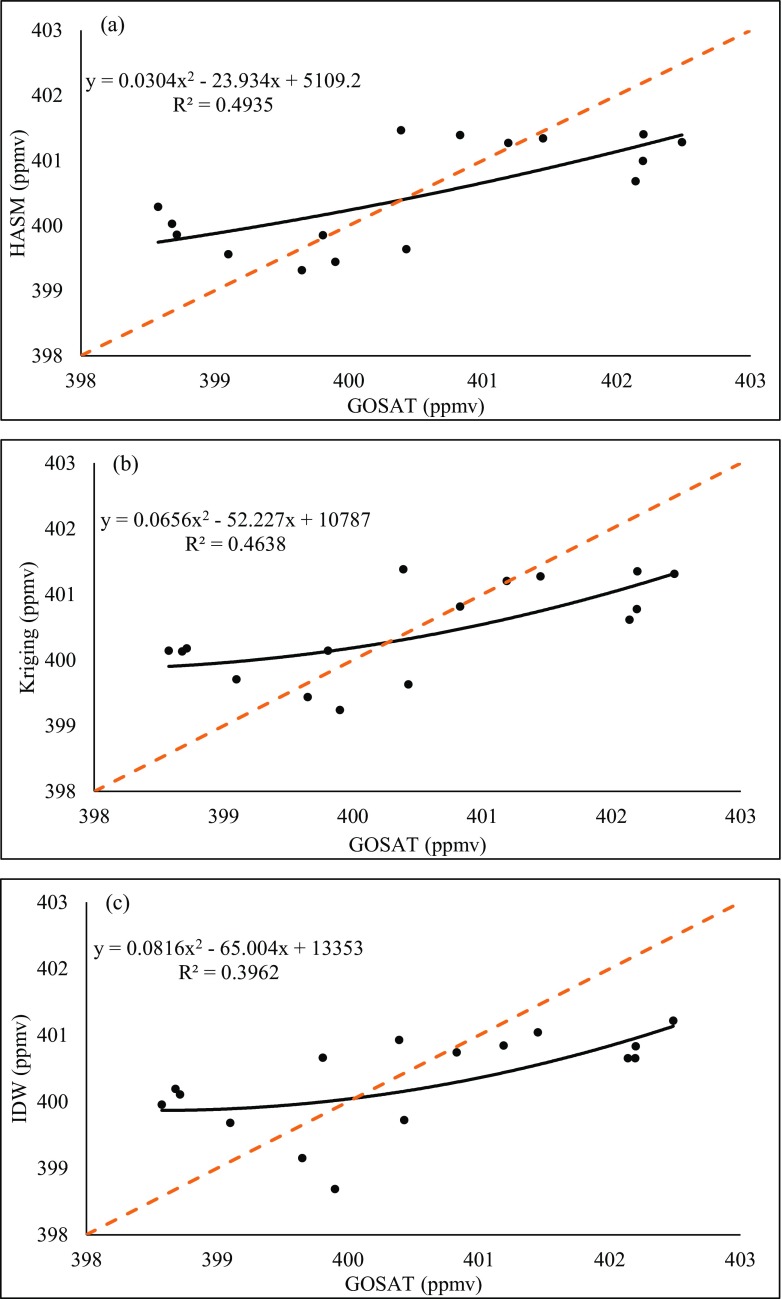
Comparison of the three methods and GOSAT retrieval data on 2015.04. **a** HASM. **b** Kriging. **c** IDW

**Fig. 8:**
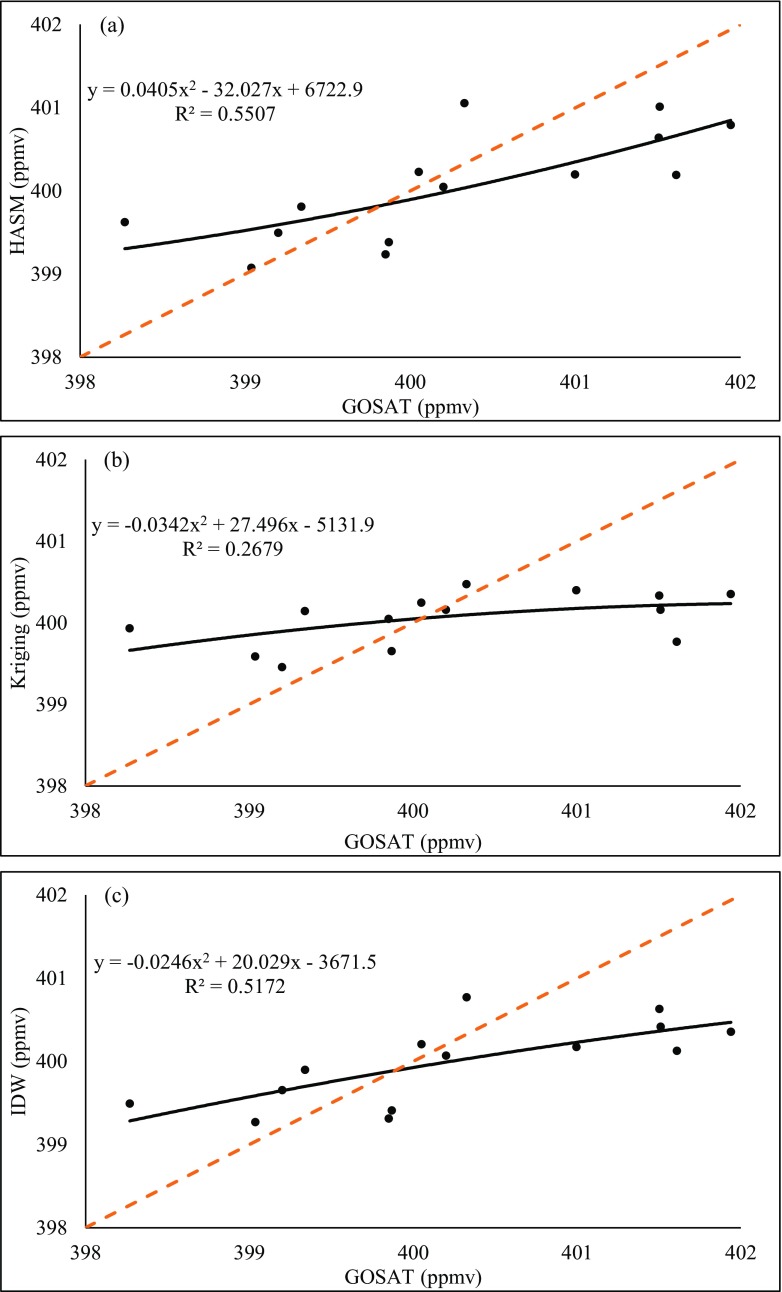
Comparison of the three methods and GOSAT retrieval data on 2015.05. **a** HASM. **b** Kriging. **c** IDW

Comparisons of the three methods and the GOSAT retrieval data for each month are shown in Figs. 5, 6, 7, and 8. The fitting equations of HASM present the highest *R*^2^ values among the three methods in all cases; meanwhile, IDW performs the worst, except in May. Furthermore, we use two statistics to show the differences of the three methods:

25 $$ \mathrm{MAE}=\frac{1}{N}\sum \limits_I^N\left|{O}_I-{S}_I\right| $$

$$ \mathrm{RMSE}=\sqrt{\frac{\sum_I^N{\left({O}_I-{S}_I\right)}^2}{N-1}} $$

Table 2 shows the monthly mean absolute error (MAE) and root mean square error (RMSE) values of the three methods and indicates that HASM perform better than the classic interpolation methods. This is because that the HASM used in this paper is not a typical spatial interpolation method to construct new data points using only a finite set of known data points. The output of HASM normally contains information from both finite known data points and an approximate field. To some extent, the HASM used in this paper is more of a data fusion method.

**Table 2:** Mean absolute error (MAE) and root mean square error (RMSE) values of the three methods

	MAE (ppmv)			RMSE (ppmv)		
	HASM	Kriging	IDW	HASM	Kriging	IDW
2015.02	1.50	1.75	1.77	1.89	2.15	2.10
2015.03	1.53	1.48	1.48	1.98	2.00	2.04
2015.04	0.80	0.83	0.95	0.98	1.03	1.10
2015.05	0.65	0.76	0.72	0.80	1.02	0.88
mean	1.12	1.27	1.23	1.41	1.55	1.53

### Spatiotemporal distribution of XCO_2_ from HASM

**Fig. 9:**
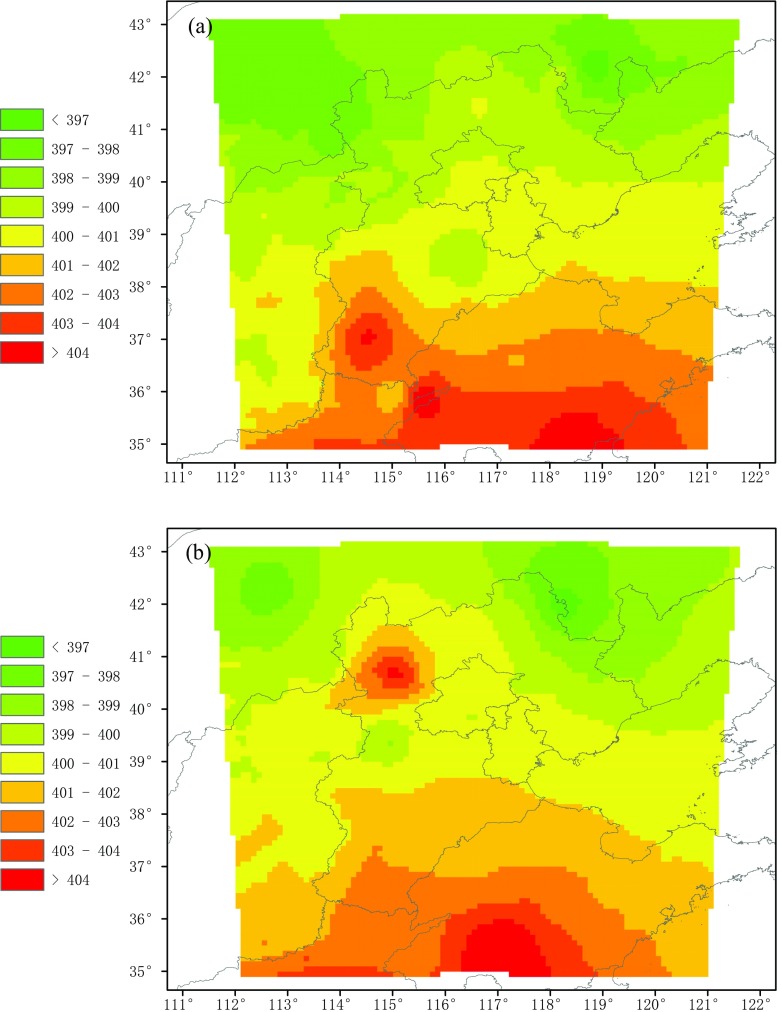

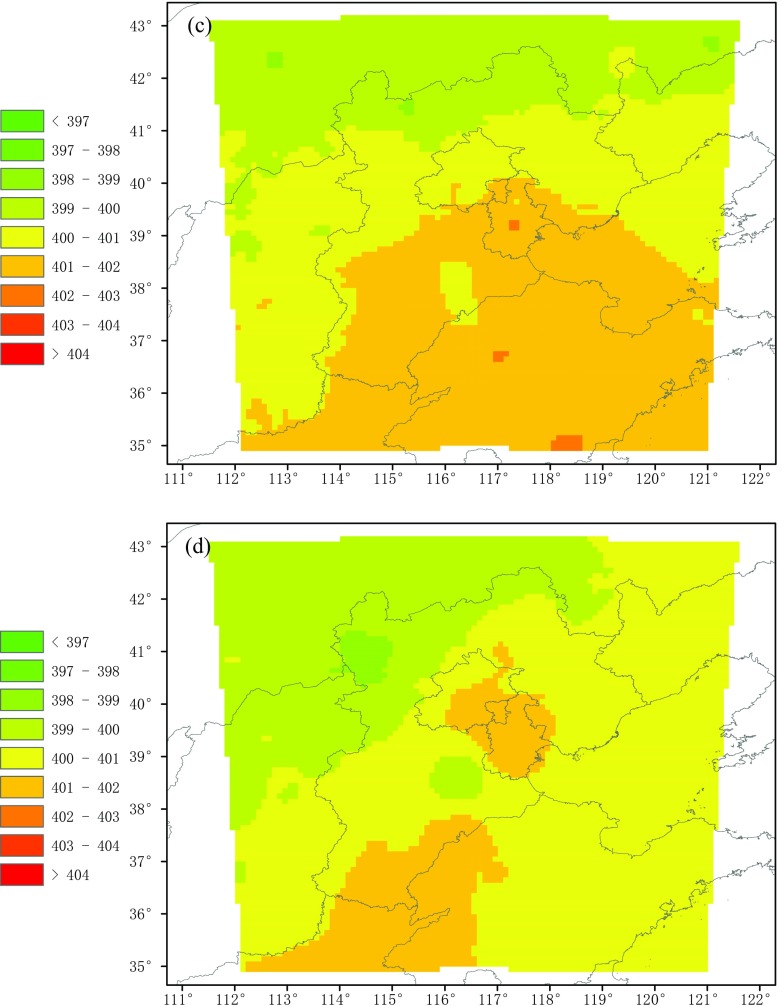
Spatiotemporal distribution of monthly XCO_2_ values from HASM. **a** February. **b** March. **c** April. **d** May

The monthly XCO_2_ outputs from HASM are reproduced in Fig. 9. The distribution of XCO_2_ varies by month. In February, the XCO_2_ is much higher in the southern regions and not the northern regions of the study area. In Shandong Province and the southern part of Hebei Province, the XCO_2_ exceeds 402 ppmv, in contrast to the values of less than 398 ppmv observed in Inner Mongolia. The spatial distribution trend in March is similar to that in February, although the high value zone contracts. This distribution phenomena may be related to the heating in the winter and the population distribution. In the northern part of study area, there is a small population and thus a lower energy consumption for heating. The middle of the study area has a great population, and thus, the XCO_2_ is relatively high. Shandong and Henan provinces are the second and third most populous in China and contribute the most carbon emission for heating. Especially in southern Shandong, burning coal to keep warm in rural areas aggravates the carbon emissions. Besides, northerly winds prevailing in the winter reinforce this distribution.

With vegetation growth in the terrestrial ecosystems and the end of heat use in April, the difference of the north-south distribution of XCO_2_ rapidly decreases, and the high value zone (i.e., greater than 402 ppmv) disappeared. The concentration is further reduced in May. Only in Beijing and Tianjin as well as at the border of Shandong, Henan, and Hebei is the XCO_2_ greater than 401 ppmv but less than 402 ppmv, which means that the carbon emissions in these areas are strongly influenced by human activity.

## Conclusions

According to the fundamental theorem of surfaces, a surface is determined by the first and the second fundamental coefficients. In this paper, HASM is applied to obtain high precision simulated XCO_2_ fields, using the simulated field outputs from the atmospheric chemical transport model as the approximate driving fields and the observed data as the accuracy control points. The main conclusions of this paper are as follows: (a) The simulation results of the regional atmospheric transport model WRF-CHEM can reflect the changes and distributions of CO_2_ concentrations to a certain extent. Although the precision of this model is poor, the result can provide driving field information for HASM for an approximate surface. (b) Comparing the output of HASM with those of the classical Kriging and IDW interpolation schemes shows that the simulation results of HASM have relatively higher accuracies during the study period. A cross-validation shows that the MAE from HASM is 1.12 ppmv and RMSE is 1.41 ppmv. c) The CO_2_ concentration field simulated by HASM has obvious spatial differentiations and changes with the seasonal changes. Due to heating emissions in the winter, densely populated areas have higher CO_2_ concentrations. With the end of the heating period and the new growth of plants, the high concentration values rapidly decrease. At this time, the distribution of high concentration area is related to industrial activities.

Compared with the previous research of HASM, the few observed data are used in this study. Meanwhile, the accuracy of the WRF-CHEM simulation field is limited. These factors will transmit biases into the outputs of HASM. Therefore, increasing the number of observation points and improving the driving field accuracies are important methods to obtain more accurate CO_2_ distributions using HASM. In addition, the GOSAT inversion data is used as the true values due to the lack of XCO_2_ observations from ground-based observation stations within the study area. This study should be repeated as more data from ground- and space-based observations become available.
